# Clinical characteristics and etiological profile of retropharyngeal space abnormalities in children: a nine-year retrospective analysis

**DOI:** 10.3389/fped.2025.1727123

**Published:** 2026-01-19

**Authors:** Lili Hao, Youhua Wei, Jiahui Lin, Juan Li, Zhongfang Xia

**Affiliations:** Department of Otaryngology, Wuhan Children’s Hospital (Wuhan Maternal and Child Healthcare Hospital), Tongji Medical College, Huazhong University of Science & Technology, Wuhan, China

**Keywords:** retropharyngeal space, deep neck infection, pediatric, congenital malformation, retropharyngeal abscess

## Abstract

**Objective:**

To analyze the clinical characteristics and etiological spectrum of retropharyngeal space abnormalities in children, stratifying by age to improve diagnostic and therapeutic strategies.

**Methods:**

We retrospectively analyzed the clinical data of 77 pediatric patients with imaging-confirmed retropharyngeal space abnormalities admitted to Wuhan Children's Hospital between January 2015 and March 2024. Patients were divided into two groups based on age: younger group (<6 years, *n* = 51) and older group (≥6 years, *n* = 26). Demographics, clinical presentations, imaging findings, etiologies, and treatment outcomes were compared between the groups using Chi-square or Fisher's exact tests for categorical data and independent samples *t*-tests for continuous data.

**Results:**

The most common presenting symptoms were fever (49.4%) and neck mass (33.8%). The primary CT finding was retropharyngeal hypodensity or fluid collection (68.8%). No significant differences were observed in sex ratio, clinical symptoms, or imaging findings between the two age groups (*p* > 0.05). However, children <6 years old had a significantly higher rate of requiring transoral incision and drainage for retropharyngeal abscess (23.5% vs. 3.8%, *p* = 0.041). Etiologically, infectious diseases were significantly more prevalent in the older group (92.3% vs. 70.6%, *p* = 0.030), whereas congenital malformations were a more common underlying cause in the younger group, however, this trend did not reach statistical significance (*p* = 0.051).

**Conclusion:**

The etiology and management of pediatric retropharyngeal abnormalities are age-dependent; infectious causes dominate in children ≥6 years, while congenital malformations are key considerations in those <6 years, who are at higher risk for abscess formation requiring surgical intervention.

## Introduction

The retropharyngeal space, a potential space in the deep neck bounded by fascial planes, is a critical anatomical region in children (1). Pathological processes within this space, though relatively uncommon, can progress rapidly to cause severe and life-threatening complications, including airway obstruction, mediastinitis, internal jugular vein thrombosis, and sepsis, posing a significant diagnostic and therapeutic challenge (2–4). The non-specific nature of early symptoms, such as fever, neck pain, and irritability, often leads to diagnostic delays, with definitive identification frequently relying on cross-sectional imaging (5).

Previous literature on pediatric deep neck pathologies has predominantly focused on pyogenic infections, particularly retropharyngeal abscesses (RPA) (6–8). While invaluable, this focus may not capture the full spectrum of conditions affecting this space. This is a crucial distinction, as the differential diagnosis includes non-suppurative inflammation (cellulitis or phlegmon), congenital lesions (e.g., branchial cleft cysts, lymphatic malformations), and inflammatory mimics such as Kawasaki disease (9, 10). Furthermore, anatomical and immunological changes during childhood are profound. The retropharyngeal lymph nodes (nodes of Rouviere), which drain the nasopharynx, adenoids, and middle ear, are prominent in early childhood but typically atrophy by age five (11, 12). This developmental anatomy suggests that the etiology and clinical course of retropharyngeal abnormalities may vary significantly with age. Despite this, comprehensive studies comparing the broad etiological landscape between different pediatric age groups remain scarce, and the epidemiology may be shifting in the era of widespread conjugate vaccination (13, 14).

This knowledge gap presents a clinical dilemma. An overemphasis on infection may lead to unnecessary surgical interventions in cases of congenital or systemic inflammatory conditions, while a failure to consider non-infectious causes in younger children can result in misdiagnosis and mismanagement (10). The primary aim of this study was therefore to investigate the etiological and clinical differences in a broad cohort of children with imaging-defined retropharyngeal space abnormalities. We hypothesized that congenital etiologies would be more prevalent in younger children (<6 years), while infectious causes would dominate in older children (≥6 years), and that these differences would correlate with distinct management requirements. By analyzing a nine-year cohort, we seek to provide clinicians with an updated, age-stratified perspective to guide more precise diagnosis and treatment.

## Materials and methods

### Patient selection and study design

This retrospective cohort study was conducted at Wuhan Children's Hospital. We reviewed the medical records of pediatric patients admitted between January 2015 and March 2024. The study protocol was approved by the Institutional Ethics Committee of Wuhan Children's Hospital (Approval No. 2024R054-E01), and the need for individual informed consent was waived due to the retrospective nature of the analysis.

**Figure 1:**
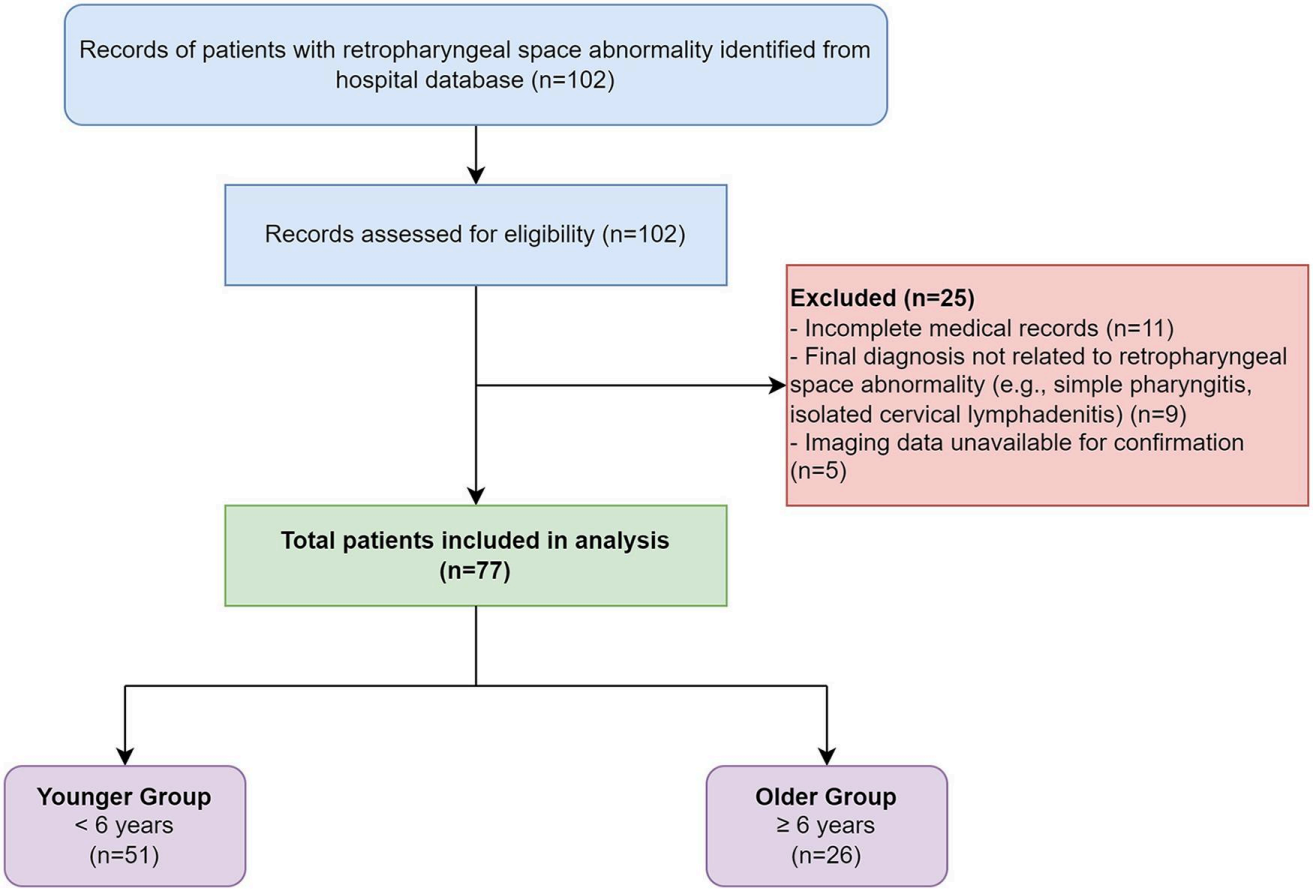
STROBE flow diagram detailing patient inclusion and exclusion criteria for the study cohort.

Inclusion criteria were: (1) age from birth to 18 years; (2) a neck computed tomography (CT) scan report explicitly mentioning abnormalities such as “retropharyngeal space infection,” “retropharyngeal fluid collection,” “retropharyngeal abscess,” or “retropharyngeal edema.” The corresponding imaging was reviewed by a senior otolaryngologist to confirm retropharyngeal soft tissue thickening (>7 mm at the C2 level or >14 mm at the C6 level) or the presence of a fluid collection or rim-enhancing lesion within the retropharyngeal space, consistent with established radiological criteria (15). The imaging review included both non-contrast and contrast-enhanced CT scans; however, definitive features such as rim-enhancing lesions required a contrast-enhanced study for confirmation. For patients with multiple admissions, only data from the first presentation were included. Patients with incomplete medical records or those with only imaging data but no clinical information were excluded. The patient selection process is detailed in Figure 1.

### Data collection and grouping

A standardized data collection form was used to extract information from the hospital's electronic medical record system. Collected data included: demographic information (age, sex), clinical presentation (including fever, neck mass, pain, and odynophagia), laboratory findings [white blood cell (WBC) count, microbial culture results], CT imaging characteristics (including retropharyngeal soft tissue thickness, presence of hypodensity, rim enhancement on contrast-enhanced scans, and cystic features), underlying or preceding diseases, treatment modalities (conservative vs. surgical), and clinical outcomes (including resolution of symptoms, need for repeat intervention, complications, and mortality). Patients were stratified into two primary groups for comparative analysis: a younger group (<6 years old) and an older group (≥6 years old). On CT imaging, “hypodensity” was defined as an area with attenuation values characteristic of fluid (approximately 0–20 Hounsfield Units, HU). A “cystic low-density lesion” was defined as a well-circumscribed, thin-walled hypodense collection. “Soft tissue density” referred to areas with attenuation similar to or greater than adjacent musculature, often indicating phlegmon or cellulitis.

### Statistical analysis

All statistical analyses were performed using SPSS software, version 25.0 (IBM Corp., Armonk, NY, USA). Continuous data are presented as mean ± standard deviation (SD) and were compared using the independent samples *t*-test. Categorical data are presented as counts and percentages (*n*, %) and were compared using the Pearson's Chi-square test or Fisher's exact test, as appropriate for expected cell counts less than 5. Odds ratios (OR) with 95% confidence intervals (CI) were calculated for key categorical outcomes. A two-tailed *p*-value of <0.05 was considered statistically significant.

## Results

### Demographic and clinical characteristics

A total of 77 patients met the inclusion criteria. The cohort included 41 males and 36 females (male-to-female ratio: 1.14:1), with a median age of 3.61 years (range: 0 days to 13.5 years). Fifty-one patients (66.2%) were in the <6 years group, and 26 patients (33.8%) were in the ≥6 years group. There was no significant difference in the sex distribution between the two age groups (*p* = 0.151).

The most common clinical presentations across the entire cohort were fever (38/77, 49.4%) and neck mass (26/77, 33.8%), followed by neck pain/swelling (16/77, 20.8%). There were no statistically significant differences in the frequencies of common symptoms between the <6 and ≥6 years groups. Other less frequent symptoms, occurring in <5% of patients, included respiratory distress, dysphagia, diarrhea, and snoring. Notably, the four neonatal cases (aged <28 days) presented differently; three were identified at birth due to a neck mass, and one developed progressive respiratory distress shortly after birth. A detailed comparison of clinical features is presented in Table 1, and representative case summaries are provided in Supplementary Table S1.

**Table 1:** Comparison of Clinical Characteristics and Management between Age Groups

Characteristic	Total (*n* = 77)	<6 years (*n* = 51)	≥6 years (*n* = 26)	*p*-value
Male sex, *n* (%)	41 (53.2)	25 (49.0)	16 (61.5)	0.151
Mean age (years), Mean ± SD	4.47 ± 3.35	2.42 ± 1.52	8.47 ± 2.08	<0.001
Clinical presentation, *n* (%)				
Fever	38 (49.4)	28 (54.9)	10 (38.5)	0.172
Neck mass	26 (33.8)	18 (35.3)	8 (30.8)	0.158
Neck pain/swelling	16 (20.8)	10 (19.6)	6 (23.1)	0.126
Neck stiffness	12 (15.6)	7 (13.7)	5 (19.2)	0.741^a^
Odynophagia	9 (11.7)	5 (9.8)	4 (15.4)	0.698^a^
Limited neck motion	4 (5.2)	2 (3.9)	2 (7.7)	1.000^a^
Other symptoms^b^	16 (20.8)	12 (23.5)	4 (15.4)	0.548^a^
Treatment modality, *n* (%)				
Conservative management only	46 (59.7)	27 (52.9)	19 (73.1)	0.088
Surgical intervention	31 (40.3)	24 (47.1)	7 (26.9)	0.088
- Transoral incision & drainage^c^	14 (18.2)	13 (25.5)	1 (3.8)	**0** **.** **041** ^a^
- Transcervical approach surgery^d^	18 (23.4)	12 (23.5)	6 (23.1)	0.880

^a^ Fisher's exact test used.

^b^ Other symptoms include respiratory distress, dysphagia, diarrhea, sore throat, parotid mass, vomiting, cough, snoring, wheezing, and skin dimple. Each occurred in <5% of the total cohort.

^c^ Includes one patient in the <6 years group who underwent both transcervical and transoral procedures.

^d^ Includes excision of congenital cysts/fistulas, drainage of associated cervical abscesses, wound debridement, and foreign body removal.

The bold values indicate statistical significance (*p* < 0.05).

### Laboratory and microbiological findings

On initial presentation, 60 patients (77.9%) had an elevated WBC count, with a mean of 19.10 ± 5.12 × 10^9^/L. Of the 17 patients with a normal WBC count, four were neonates (mean WBC: 17.48 ± 4.45 × 10^9^/L, within the normal range for this age) and 13 were older children (mean WBC: 9.67 ± 3.78 × 10^9^/L).

Microbial cultures (blood, sputum, or pus) were performed in 38 patients (49.4%). A positive result was obtained in 9 of these 38 cases (23.7%). The identified pathogens included *Streptococcus constellatus* (*n* = 3), *Streptococcus pneumoniae* (*n* = 1), and *Stenotrophomonas maltophilia* (*n* = 1). In four cases, cultures grew normal upper respiratory flora. The remaining 29 cultures yielded no growth. Among the 38 patients who had cultures performed, 25 (65.8%) had received at least one dose of antibiotics prior to sample collection.

### Imaging findings and underlying etiology

All patients had abnormalities detected on their initial CT scan. Contrast enhancement was used in 45 cases (58.4%). The most frequent CT finding was hypodensity or fluid collection in the retropharyngeal space (53/77, 68.8%), followed by soft tissue density enhancement (16/77, 20.8%) and cystic low-density lesions (6/77, 7.8%). No significant difference was observed in the distribution of these CT findings between the two age groups (Table 2). Of the 45 patients who received intravenous contrast, 38 (84.4%) demonstrated peripheral rim enhancement around a central hypodense collection, a finding strongly suggestive of abscess formation. The presence of rim enhancement was noted in 28 of the 33 younger patients (84.8%) and 10 of the 12 older patients (83.3%) who received contrast, with no significant difference between the groups (*p* = 0.915). No lesions were found to be hyperdense on non-contrast scans suggestive of proteinaceous material. One case in the ≥6 years group, associated with a traumatic foreign body, showed a focus of intense, solid-appearing enhancement within the phlegmonous tissue.

**Table 2:** Comparison of Etiology and CT Findings between Age Groups

Finding	Total (*n* = 77)	<6 years (*n* = 51)	≥6 years (*n* = 26)	*p*-value
Primary CT description, *n* (%)				
Hypodensity/fluid collection	53 (68.8)	33 (64.7)	20 (76.9)	0.267
Soft tissue density	16 (20.8)	13 (25.5)	3 (11.5)	0.154^a^
Cystic hypodensity	6 (7.8)	6 (11.8)	0 (0.0)	0.091^a^
Other^b^	2 (2.6)	0 (0.0)	2 (7.7)	0.130^a^
Underlying etiology, *n* (%)				
Infectious diseases	**60** **(77.9)**	**36** **(70.6)**	**24** **(92.3)**	**0** **.** **030** ^a^
- Lymphadenitis	35 (45.5)	23 (45.1)	12 (46.2)	
- Cervical abscess	12 (15.6)	6 (11.8)	6 (23.1)	
- Sepsis	9 (11.7)	6 (11.8)	3 (11.5)	
- Peritonsillar abscess	4 (5.2)	1 (2.0)	3 (11.5)	
Congenital malformations	**12** **(15.6)**	**11** **(21.6)**	**1** **(3.8)**	**0** **.** **051** ^a^
- Lymphatic malformation	5 (6.5)	5 (9.8)	0 (0.0)	
- Branchial cleft anomaly	6 (7.8)	5 (9.8)	1 (3.8)	
-Lingual root cyst	1 (1.3)	1 (2.0)	0 (0.0)	
Immunological/inflammatory	**3** **(3.9)**	**2** **(3.9)**	**1** **(3.8)**	**1** **.** **000** ^a^
Other (trauma/foreign body)	**2** **(2.6)**	**0** **(0.0)**	**2** **(7.7)**	**0** **.** **130** ^a^

Percentages for subgroups are based on the column total. *P*-values are for the main category comparisons.

^a^ Fisher's exact test used.

^b^ Includes gas density and high density (foreign body).

The bold values indicate statistical significance (*p* < 0.05).

Repeat imaging was performed in 22 patients (28.6%) due to lack of clinical improvement or to assess post-operative changes. This was more frequent in the younger group, with 18 of 51 patients (<6 years, 35.3%) undergoing repeat scans compared to 4 of 26 in the older group (≥6 years, 15.4%). An exploratory analysis revealed that an initial retropharyngeal soft tissue thickness greater than 10 mm at the C2 vertebral level was significantly associated with the need for subsequent imaging (*p* = 0.021).

**Figure 2:**
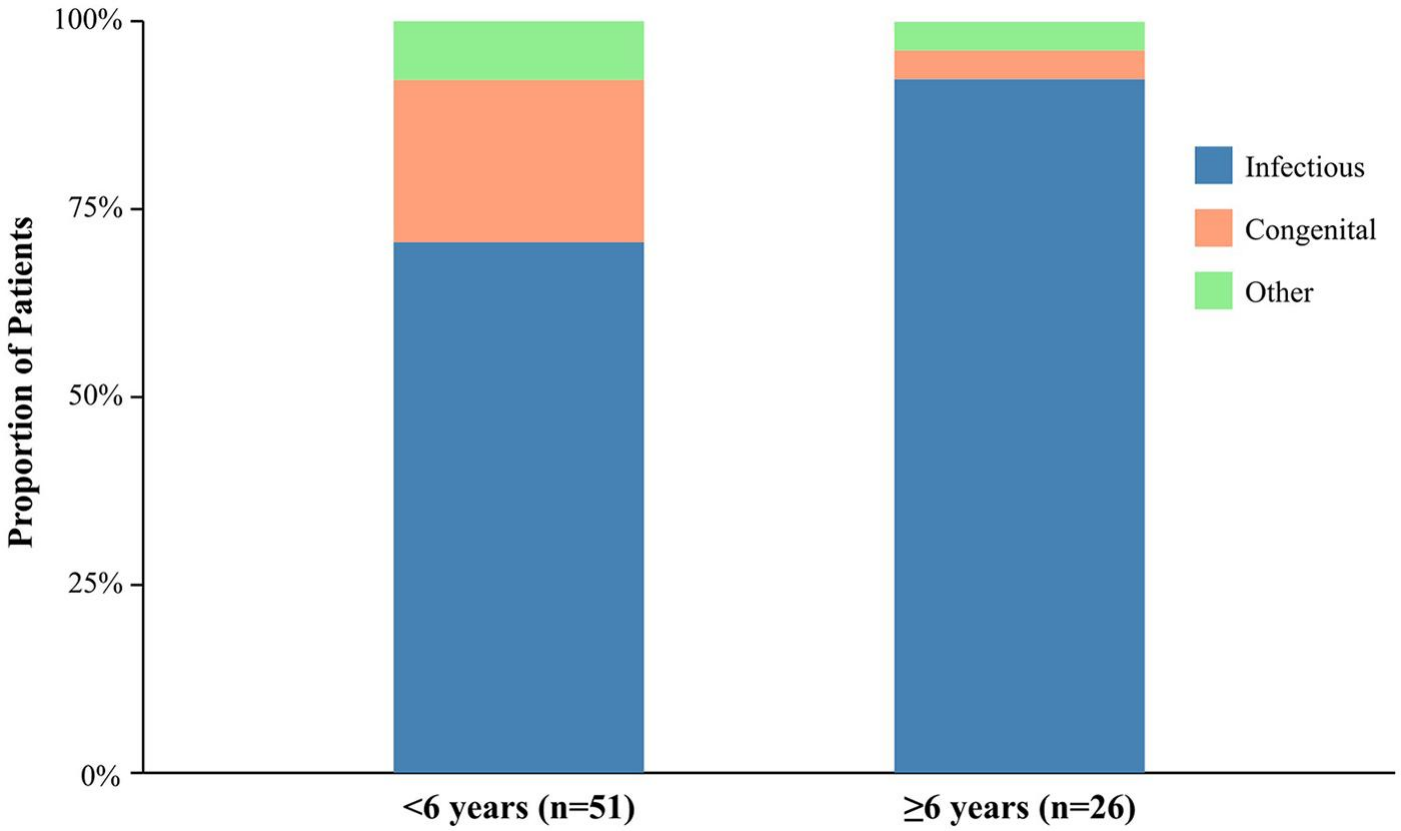
Stacked bar chart illustrating the distribution of underlying etiologies for retropharyngeal space abnormalities between the two pediatric age groups. A clear shift from a mixed etiological profile in younger children to a predominantly infectious profile in older children is evident (*p* = 0.030 for infectious diseases).

The underlying or concurrent diseases were categorized as infectious, congenital, immunological, or other (e.g., trauma). As shown in Table 2 and Figure 2, the etiological profile differed significantly between age groups. Infectious diseases were the predominant cause in the ≥6 years group, accounting for 92.3% of cases, which was significantly higher than the 70.6% observed in the <6 years group (OR: 5.33, 95% CI: 1.15–24.7, *p* = 0.030). Conversely, congenital malformations (including lymphatic malformations and branchial cleft anomalies) were identified as an underlying cause more frequently in children <6 years old (21.6%) compared to the ≥6 years group (3.8%); however, this trend did not reach statistical significance (*p* = 0.051).

### Treatment and outcomes

Overall, 46 patients (59.7%) were successfully managed with conservative treatment alone, which typically consisted of intravenous antibiotics (amoxicillin-clavulanate or a first-generation cephalosporin), metronidazole, and corticosteroids. The remaining 31 patients (40.3%) required surgical intervention. The rate of conservative treatment was higher in the ≥6 years group (73.1%) compared to the <6 years group (52.9%), but this difference was not statistically significant (*p* = 0.088).

A key difference emerged in the type of surgical intervention required. The rate of transoral incision and drainage, indicative of a true retropharyngeal abscess, was significantly higher in the <6 years group (13/51, 25.5%) compared to the ≥6 years group (1/26, 3.8%) (OR: 8.55, 95% CI: 1.05–69.8, *p* = 0.041). Two patients required a second drainage procedure. All patients recovered fully and were discharged without major complications such as airway compromise requiring tracheotomy. No mortality was recorded.

## Discussion

This nine-year retrospective study provides a comprehensive analysis of retropharyngeal space abnormalities in a pediatric cohort, revealing significant age-related differences in etiology and management. Our primary findings confirm the hypothesis that while infectious causes are ubiquitous, congenital malformations are a crucial etiological factor in children under six, who are also more likely to require surgical drainage for abscess formation.

Consistent with previous reports, our study found fever and neck mass to be the most common presenting signs (6, 16). The non-specific nature of these symptoms, especially in non-verbal younger children, underscores the diagnostic challenge. While CT with contrast remains the gold standard for diagnosis, differentiating cellulitis from a drainable abscess and identifying underlying structural anomalies (17–19), its interpretation must be integrated with the patient's age and clinical context. For instance, some evidence suggests that ultrasound can be a valuable adjunct, particularly for follow-up and avoiding repeated radiation exposure in children (20). Our findings showed that over two-thirds of patients presented with hypodensity or fluid, but this finding alone was not discriminative between age groups, reinforcing the need for nuanced clinical-radiological correlation.

The most significant finding of our study is the clear etiological shift that occurs around the age of six. In children ≥6 years, nearly all cases (92.3%) were secondary to infection, typically originating from pharyngotonsillitis or cervical lymphadenitis, a pattern consistent with established pathophysiology (21). In contrast, children <6 years presented a more diverse etiological profile. A substantial proportion (21.6%) had underlying congenital malformations, such as infected second branchial cleft cysts or lymphatic malformations extending medially (22, 23). This finding is clinically paramount. The prominence of retropharyngeal lymph nodes in infancy makes this group susceptible to primary suppurative lymphadenitis (11), while concurrently, congenital remnants are more likely to first manifest or become infected during these early years (1, 24). This dual susceptibility requires clinicians to maintain a high index of suspicion for congenital causes, which may necessitate more complex surgical planning beyond simple drainage (23). The trend towards a higher prevalence of congenital malformations in the younger group (*p* = 0.051), while not reaching statistical significance, may be clinically important and could reflect a type II error due to our sample size.

Our study supports the prevailing consensus that many deep neck inflammatory conditions can be managed conservatively (25, 26). Nearly 60% of our cohort recovered without surgery. However, the need for intervention was strongly associated with age. The significantly higher rate of transoral incision and drainage in the <6 years group directly reflects a higher propensity for abscess formation. This is likely due to the robust and easily infected lymphatic tissue in the retropharyngeal space of younger children (11, 27). This finding serves as a practical guide: while a trial of conservative therapy is appropriate, clinicians should have a lower threshold for surgical drainage in febrile infants and young children with a well-defined retropharyngeal fluid collection, especially if it exceeds 2 cm in diameter (17, 28, 29).

Our results resonate with recent literature. The study by Harounian et al. (2018), which focused on cervical infections in infants vs. older children, similarly found that infants were significantly more likely to require surgical intervention despite presenting with fewer and more subtle clinical symptoms (30). Furthermore, Ratnapalan et al. (2025) conducted a large review of retropharyngeal and parapharyngeal infections, highlighting that children under 12 months were sicker at presentation and had a markedly higher complication rate (31). Our study complements this work by focusing on a different age stratification (<6 vs. ≥6 years) that aligns with the known involution of retropharyngeal lymph nodes and uniquely emphasizes the significant role of underlying congenital anomalies as a predisposing factor in the younger cohort, a factor not deeply explored in the other studies.

The low microbial yield in our study (23.7%) is a common finding in deep neck infection literature and is largely attributable to prior antibiotic administration (7, 32). The identified pathogens, primarily *Streptococcus* species, are consistent with typical oropharyngeal flora. However, it is crucial to recognize that deep neck infections are often polymicrobial, involving anaerobes that may not be recovered with standard culture techniques (21). Empiric antibiotic therapy should therefore provide broad coverage for Gram-positive cocci, respiratory pathogens, and anaerobes, with consideration for Methicillin-resistant *Staphylococcus aureus* (MRSA) in communities with high prevalence (33).

An important finding was the two cases of Kawasaki disease (KD) in the <6 years group, which presented with retropharyngeal edema and persistent fever. Initially managed for a presumed abscess, they were diagnosed with KD post-operatively when fever persisted despite drainage yielding only serous fluid. This highlights the critical importance of including KD in the differential diagnosis of retropharyngeal edema in febrile children (9, 10). Radiologically, KD-associated retropharyngeal edema is typically diffuse and non-rim-enhancing, distinguishing it from a mature abscess (34). Recognizing this “abscess-like” presentation of KD is vital to prevent unnecessary surgery and to allow prompt initiation of intravenous immunoglobulin (IVIG) therapy, the cornerstone of treatment to prevent coronary artery aneurysms (34, 35).

Ultimately, these findings underscore the need for a prospective, multi-center registry with standardized imaging, microbiological sampling, and treatment protocols to develop more definitive, evidence-based management guidelines for this condition.

## Limitations

This study has several limitations. Its retrospective design is subject to selection bias and reliance on the accuracy of medical records. As a single-center study, our findings may not be fully generalizable. Furthermore, inherent retrospective data collection means that details regarding CT acquisition parameters (e.g., scanner vendor, kVp, mAs, contrast timing), sedation rates, and radiation dose (DLP/CTDIvol) were not standardized or available for analysis. The low microbial yield, compounded by prior antibiotic use, limits our ability to characterize the bacteriology definitively. We also lacked uniform protocols for microbial sampling (e.g., site, timing relative to antibiotics, use of anaerobic cultures or nucleic acid amplification tests). Finally, our statistical analysis was limited to unadjusted comparisons due to the sample size, precluding multivariable logistic regression or sensitivity analyses (e.g., excluding neonates or varying abscess size thresholds) that could have controlled for confounding variables. Therapeutic protocols may also have evolved over the nine-year study period.

## Conclusion

**Figure 3:**
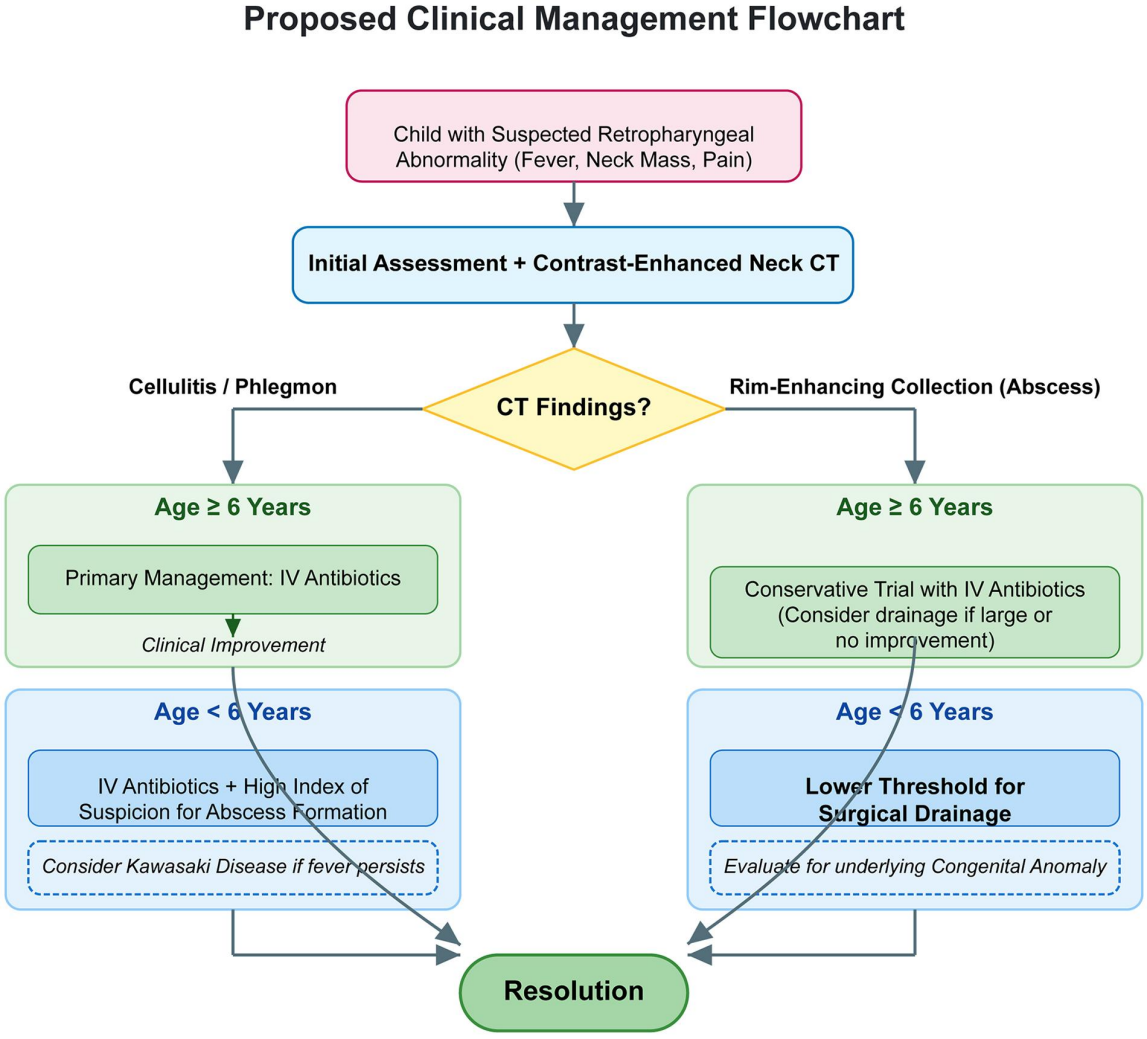
Proposed clinical management flowchart for pediatric retropharyngeal space abnormalities. The algorithm outlines a step-by-step, age-stratified approach from initial presentation and imaging to definitive management based on the primary findings of this study.

Based on our findings, we propose a clinical management approach for pediatric retropharyngeal abnormalities that is stratified by age. For any child presenting with signs of deep neck infection, contrast-enhanced CT is the cornerstone of diagnosis to differentiate cellulitis from a drainable abscess. In children ≥6 years, where the etiology is predominantly infectious, a trial of conservative management with intravenous antibiotics is the preferred initial step for cellulitis or small collections. In contrast, for children <6 years, a lower threshold for surgical intervention is warranted due to their higher propensity for abscess formation. Furthermore, in this younger cohort, clinicians must maintain a high index of suspicion for underlying congenital anomalies and consider Kawasaki disease in cases of non-suppurative edema with persistent fever. A summary of this proposed step-by-step clinical pathway is presented in Figure 3.

## Data Availability

The original contributions presented in the study are included in the article/Supplementary Material, further inquiries can be directed to the corresponding author.
