# Supplementary material for: Clinical characteristics and etiological profile of retropharyngeal space abnormalities in children: a nine-year retrospective analysis
Source: Front Pediatr. 2026 Jan 19;13:1727123. doi: 10.3389/fped.2025.1727123 (PMC12862053; doi:10.3389/fped.2025.1727123)
Supplement: Supplementary file 1 [file Table1.docx]

**Supplementary Table 1. Representative Case Summaries**

| **Characteristic** | **Case 1** | **Case 2** | **Case 3** | **Case 4** |
| --- | --- | --- | --- | --- |
| **Age/Sex** | 25 days / Male | 3 years / Female | 8 years / Male | 4 years / Male |
| **Presentation** | Neck mass since birth, progressive feeding difficulty, intermittent stridor. Afebrile. | 5-day history of high fever (39.5°C), neck pain, torticollis, and drooling. | 3-day history of fever, sore throat, and right-sided neck swelling. No respiratory distress. | 6-day history of persistent high fever, bilateral conjunctivitis, rash, and tender cervical mass. |
| **Key Lab Finding** | WBC: 16.5 x 10^9^/L | WBC: 22.8 x 10^9^/L, CRP: 150 mg/L | WBC: 18.2 x 10^9^/L, CRP: 95 mg/L | WBC: 20.5 x 10^9^/L, CRP: 220 mg/L, elevated ESR. |
| **CT Findings** | Large, multi-loculated cystic hypodense lesion in the left neck extending into the retropharyngeal space. No significant rim enhancement. | Large (2.5 cm diameter) rim-enhancing hypodense collection in the retropharyngeal space with significant mass effect on the airway. | Diffuse retropharyngeal and right parapharyngeal soft tissue thickening and enhancement, consistent with cellulitis. No drainable collection. | Diffuse retropharyngeal edema without a rim-enhancing collection. Prominent cervical lymphadenopathy. |
| **Final Diagnosis** | Infected Cervical Lymphatic Malformation | Retropharyngeal Abscess | Retropharyngeal Cellulitis | Kawasaki Disease |
| **Management** | Surgical excision via transcervical approach. IV antibiotics. | Transoral incision and drainage. IV ampicillin-sulbactam. | IV ceftriaxone and clindamycin. No surgery. | IV Immunoglobulin (IVIG) and high-dose aspirin. No surgery. |
| **Outcome** | Full recovery, discharged on day 10. | Fever resolved within 24 hours post-op. Discharged on day 5. | Symptoms improved within 48 hours. Discharged on day 4. | Fever resolved post-IVIG. Discharged on day 7 with follow-up echocardiogram. |
